# Inventory-transportation integrated optimization for maintenance spare parts of high-speed trains

**DOI:** 10.1371/journal.pone.0176961

**Published:** 2017-05-04

**Authors:** Boliang Lin, Jiaxi Wang, Huasheng Wang, Zhongkai Wang, Jian Li, Ruixi Lin, Jie Xiao, Jianping Wu

**Affiliations:** 1 School of Traffic and Transportation, Beijing Jiaotong University, Beijing, People's Republic of China; 2 Locomotive & Car Research Institute, China Academy of Railway Sciences, Beijing, People's Republic of China; 3 Institute of Computer Technologies, China Academy of Railway Sciences, Beijing, People's Republic of China; 4 Department of Electrical Engineering, Stanford University, Stanford, California, United States of America; Lanzhou University of Technology, CHINA

## Abstract

This paper presents a 0–1 programming model aimed at obtaining the optimal inventory policy and transportation mode for maintenance spare parts of high-speed trains. To obtain the model parameters for occasionally-replaced spare parts, a demand estimation method based on the maintenance strategies of China’s high-speed railway system is proposed. In addition, we analyse the shortage time using PERT, and then calculate the unit time shortage cost from the viewpoint of train operation revenue. Finally, a real-world case study from Shanghai Depot is conducted to demonstrate our method. Computational results offer an effective and efficient decision support for inventory managers.

## Introduction

As an energy-saving and environmentally friendly transportation mode, railways have attracted much attention in recent years. The railway network, especially the high-speed railway (HSR), has developed rapidly in China. For example, China had built a HSR network of 19,000 km by the end of 2015 [1]. According to the long-term plan released recently, the HSR network size is expected to reach 30,000 km in 2020 and 38,000 km in 2025 [2]. Along with construction of the HSR network, the demand for high-speed trains is increasingly strong. At present, China’s railway owns 2,395 electric multiple units (EMU) trains, providing more than 4,200 high-speed train services and delivering more than 4,000,000 passengers every day on the HSR network. Ever since the Beijing-Tianjin high-speed rail line (which is generally regarded as the first high-speed line in mainland China) was put into operation in 2008, the accumulative mileage of all high-speed EMU trains has reached more than 3.74 billion km and a considerable number of EMU trains have reached their overhaul cycles [3]. Therefore, the ordering and inventory of overhaul spare parts for high-speed trains have been placed on the agenda.

### Motivations

It is well known that high-speed EMU trains should be inspected and maintained after a pre-defined travel distance for safety reasons. During the maintenance process, some worn components are replaced. Thus, EMU depots (where EMU trains are overhauled) should not only provide a range of inspection equipment but also stock a large number of spare parts. Clearly, excess inventory of spare parts will lead to high holding costs. In contrast, insufficient inventory yields a low service level and may impact on the maintenance process. Worse yet, a shortage of spare parts may delay putting the EMU trains into service on schedule, which can lead to vast economic losses [4, 5]. Hence, determining how many spare parts should be stored in EMU depots has become an urgent scientific problem for the high-speed railway system [6]. This issue is even trickier for occasionally-replaced spare parts (OSP) that are required within an EMU overhaul process because their demand is highly random, with little historical data and lacking in statistical samples.

Transportation plays a major role in supply chain management. If the transportation costs of EMU spare parts are low, and the parts can be delivered in time, a zero inventory strategy is worth pursuing. However, uncertainty always exists in the transportation and delivery process, and as a result, transportation time windows need to be considered when developing inventory strategies in practice. As inventory and transportation are highly correlated [7], their joint optimization represents a critical and important task for inventory managers.

### Related work

#### Spare parts: Demand forecasting

Demand for spare parts is typically intermittent and lumpy, and forecasting the relevant requirements constitutes a very challenging exercise [8]. Croston [9] described a method of forecasting intermittent demand by using separate estimates of the size of demand, and of the demand frequency. The rules for setting the safety stock levels had also to be adjusted before consistent protection could be obtained against being out of stock. However, Croston’s method has the disadvantages of biased estimation and obsolescence. Kamath and Pakkala [10] outlined a Bayesian approach to demand estimation for the cases of stationary as well as non-stationary demand, which was particularly useful for long planning horizons. Moreover, the bootstrap procedure was also widely used to address the intermittent demand [11].

In early literature, the demand of spare parts was primarily assumed to be normally distributed [9], geometrically distributed [12], or a gamma distribution [13]. However, these assumptions lack of empirical support. Syntetos et al. [14] conducted a detailed empirical investigation on the goodness-of-fit of various distributions and their stock-control implications in terms of inventories held and service levels achieved. The results of their empirical investigation suggest that the negative binomial distribution (NBD) performs best in an inventory context, followed by the Gamma and Stuttering Poisson distribution.

Recently, maintenance policies have been considered when forecasting spare parts demand. On this basis, integrated demand forecasting and equipment maintenance models are proposed. Wang and Syntetos [8] presented a novel idea to forecast service parts demand that relies upon the very sources of the demand generation process and compared it with a well-known time-series method. Subsequently, Wang [15] presented the joint optimization for both the inventory control of the spare parts and the preventive maintenance inspection interval. Romeijnders et al. [16] proposed a two-step method for forecasting spare parts demand by separately updating the average number of parts needed per repair and the number of repairs for each type of component. These maintenance-driven models provide better accuracy than traditional methods, and thus they are recommended for practical applications.

#### Spare parts: Inventory policies

The inventory control policies need to be developed once the spare parts demand is obtained. Because spare parts inventories differ from those of work-in-process and finished products, unique aspects of maintenance inventories have been considered in previous studies (see the overview by [17]). Kocaga and Sen [18] studied an inventory system that consisted of two demand classes. The orders in the first class needed to be satisfied immediately, whereas the orders in the second class were to be filled in a given demand lead time. Selcuk [19] presented an adaptive base stock policy for a repairable item inventory control problem. van Jaarsveld et al. [20] investigated spare parts inventory control at a repair shop, and found that performance of the repair shop should be measured on the level of component repairs, rather than on the level of spare parts. A binary programming model was proposed and then an automated approach was presented to solve the model, in which each combination of policy parameters (*s*, *S*) for a spare part was represented by a column. Guajardo et al. [21] focused on how to set control parameters for the (*s*, *S*) continuous review system, subject to the fill rate service-level constraint. The fitness of seven demand models to the data of about 21,000 items were studied. Topan et al. [22] proposed a heuristic that was efficient and tractable for real-life problems with large numbers of items. They also proved that the heuristic was asymptotically optimal in the number of parts. However, the above literature do not consider the transportation process in the supply chain management. Indeed, impacts of transportation on the development of inventory policy lie on various aspects. For example, since different transportation modes correspond to different delivery time, to ensure the buyer can receive items within a given time window (e.g. the lead time), a reasonable transportation mode has to be selected. Otherwise, it may result in extra holding costs or shortage costs.

Compared with independent inventory optimization, integrated inventory-transportation related considerations of the spare parts inventory problem is an area that has not received sufficient attention in the literature. Qu et al. [23] developed an integrated inventory-transportation system with a modified periodic-review inventory policy and a travelling-salesman component. Cardós and García-Sabater [24] integrated inventory and transportation decisions into a single model where transportation costs were calculated on the basis of detailed delivery schedules. Kutanoglu and Lohiya [5] presented an optimization-based model to gain insights into the integrated inventory and transportation problem for a single-echelon, multi-facility service parts logistics system with time-based service level constraints. Zhao et al. [25] introduced a vendor-managed inventory concept to address integrated inventory and transportation problem under the scenario of a petroleum and chemical corporation in China, with its subsidiaries along the Yangtze River. Jha and Shanker [26] investigated an integrated inventory problem with transportation in a single-vendor and multi-buyer divergent supply chain. The issue of transportation was addressed through vehicle routing in the model. The possibility of the buyers’ lead time reduction had also been incorporated in the study in the environment of independent normally distributed external demand on the buyers. Amini and Ghodsi [7] addressed an integrated transportation and inventory problem in a two-stage supply chain, including suppliers and retailers, while considering the role of energy in terms of fuel's type selection. Lee et al. [27] developed an integrated single-vendor multi-buyer inventory-transportation supply chain model by adding the shipment and delivery cost to the synchronized cycles model. However, above methods cannot be applied to our work directly since unique aspects should be considered in a high-speed railway system.

### Contributions and paper organization

Despite relatively rich literature on service spare parts demand forecasting and inventory policy development, few studies involve high-speed train maintenance parts. Some unique problems should be carefully examined for these parts when developing inventory policies, such as how to calculate the shortage cost caused by delay of train services and how to take into consideration of train maintenance strategies. In this paper, we address the inventory-transportation integrated optimization problem from both aspects of theoretical analysis and practical application. Our contributions can be summarized as follows:

1. A 0–1 programming model is constructed to address the integrated inventory and transportation mode selection optimization problem aimed at achieving the minimal costs.
2. We present a novel demand estimation method for EMU occasionally-replaced spare parts on the basis of the maintenance strategies of China’s high-speed railway system.
3. A framework for calculating the shortage cost of EMU spare parts is proposed. This framework includes two aspects, i.e., using PERT to calculate the shortage time and estimating the shadow loss of absence from train operation service in a single day.
4. We demonstrate our method with a real-world instance from Shanghai Depot by an enumeration technique, and the computational results can provide the depot with an effective decision support.

The remainder of this paper is organized as follows. Section 2 presents the 0–1 programming model to address the integrated inventory-transportation problem, together with analysis of total costs of two basic inventory policies. Section 3 proposes a novel demand estimation method for EMU OSP based on maintenance strategies of China high-speed railway system. Section 4 analyzes the shadow loss of absence from train operation service caused by the shortage of spare parts using PERT. Section 5 demonstrates our method by a real-world example. Finally, conclusions and directions for future research are discussed in Section 6.

## The 0–1 programming model for integrated inventory-transportation optimization

There are various types of inventory policy, such as the reorder point policy, the fixed-time period method, and the just-in-time strategy. These policies can be roughly divided into two basic types: the advance order policy (AOP) and the temporary order policy (TOP). The advance order policy states that in order to avoid being out of stock, items are stored in warehouses in advance, on the basis of materials consumption law. In contrast, under the temporary order policy, items are ordered when needed. For each inventory policy, we consider three major transportation modes: rail, truck, and air transport. The aim of our study is to determine the optimal (the most economical) inventory policy as well as the optimal transportation mode. In this section, we propose a 0–1 programming model to address this integrated inventory-transportation optimization problem.

### Model formulation

In our model, the following notations are used.

#### Sets and indices

*M*: set of transportation modes, indexed by *m* = 1,2,3 (1: rail, 2: truck, 3: air)

#### Decision variables

*x*: binary variable, it takes a value of 1 when the AOP is adopted, otherwise, when TOP is adopted, it takes a value of 0
*y_m_*: binary variable, it takes a value of 1 when transportation mode *m* is adopted, otherwise, it takes a value of 0

#### Cost parameters

*f*(*y*_*m*_): total cost of the AOP with transportation mode *m*
*f*′(*y*_*m*_): total cost of the TOP with transportation mode *m*

Intended to achieve the minimal costs of inventory, the integrated inventory-transportation problem can be formulated as a 0–1 programming model as follows:

$$\text{Minimize }f(y_{m})\cdot x+f^{\prime }(y_{m})\cdot (1-x)$$ (1)

$$\text{Subject to }\sum_{m\in M}y_{m}=1$$ (2)

x∈{0,1} (3)

$$y_{m}\in \left\{0,1\right\}\;m=1,2,3$$ (4)

Objective Function (1) is the total costs of inventory. Constraint (2) ensures that each inventory policy can only adopt one transportation mode. Constraints (3) and (4) are the binary restrictions on decision variables.

### Inventory costs of the AOP

Under the AOP, we consider the following costs: ordering cost, purchase price, inventory holding cost, transportation cost and in-transit inventory cost. In this way, the total cost (derived from [28]) of the AOP with transportation mode *m* can be expressed as follows.

$$f(y_{m})=\frac{D}{\sum_{m}Q_{m}y_{m}}\sum_{m}C_{m}^{\text{Order}}y_{m}+D\sum_{m}C_{m}^{\text{Transport}}y_{m}+\frac{\gamma PD}{365}\sum_{m}T_{m}^{\text{Tranport}}y_{m}+PD+(I^{\text{Safety}}+\frac{1}{2}\sum_{m}Q_{m}y_{m})\left[C^{\text{Stock}}+(P+\sum_{m}C_{m}^{\text{Transport}}y_{m})\gamma \right]$$ (5)

where *D* is the annual demand, $C_{m}^{\text{Order}}$ is the ordering cost with mode *m*, *Q*_*m*_ is the order quantity with mode *m*, $C_{m}^{\text{Transport}}$ is the unit transportation cost, *γ* is the interest rate per year, *P* is the unit purchase price, $T_{m}^{\text{Tranport}}$ is the transportation time with mode *m* in days, *I*^Safety^ is safety stock, and *C*^Stock^ is the storage cost per unit. The five terms on the right side of Eq (5) are ordering cost, transportation cost, in-transit inventory cost, annual purchase cost, and annual holding cost, respectively.

### Inventory costs of the TOP

In the TOP, items are ordered when they are needed; that is, there are no holding costs. However, stockouts may occur as the transportation process can sometimes be out of the time window. In fact, the TOP avoids the holding costs at the price of shortage costs.

Similar to the AOP, the total cost of the TOP with transportation mode *m* can be expressed as follows.

$$f^{\prime }(y_{m})=\frac{365}{T^{\text{Interval}}}\sum_{m}C_{m}^{\text{Order}}y_{m}+D\sum_{m}C_{m}^{\text{Transport}}y_{m}+\frac{\gamma PD}{365}\sum_{m}T_{m}^{\text{Transport}}y_{m}+PD+C^{\text{Shortage}}\sum_{m}T_{m}^{\text{Shortage}}y_{m}$$ (6)

where *T*^Interval^ is the average reorder interval, *C*^Shortage^ is unit time shortage cost, and $T_{m}^{\text{Shortage}}$ is the shortage time. Please note that both annual purchase cost terms in the AOP and TOP are identical, which means they have no effects on the optimization process. However, to highlight the total costs of inventory and without loss of generality, we still preserve the annual purchase cost term in the computational process.

### Solution framework

Clearly, there are six candidate solutions of the 0–1 programming model, which means we can apply an enumeration method to solve it. In fact, for the AOP, without any doubt, we would adopt the least costly transportation mode; that is, the rail transport mode. As a result, the following four candidate solutions (**X**_**1**_ ~ **X**_**4**_) need to be considered: **X**_**1**_ = {*x* = 1, *y*_1_ = 1, *y*_2_ = 0, *y*_3_ = 0}, **X**_**2**_ = {*x* = 0, *y*_1_ = 1, *y*_2_ = 0, *y*_3_ = 0}, **X**_**3**_ = {*x* = 0, *y*_1_ = 0, *y*_2_ = 1, *y*_3_ = 0}, and **X**_**4**_ = {*x* = 0, *y*_1_ = 0, *y*_2_ = 0, *y*_3_ = 1}.

To calculate the total costs of inventory for each candidate solution, we need to first obtain the annual demand. To this end, we describe the detailed demand estimation method in Section 3. In addition, in the TOP, the shortage costs should also be obtained. With respect to this point, a PERT is used to calculate the shortage costs from the perspective of train operation revenue in Section 4. Note that the order quantity *Q*_*m*_ in Eq (5) is unknown. To achieve minimal costs, we use the EOQ model to determine the optimal lot size:

$$Q^{*}=\sqrt{\frac{2D\sum_{m}C_{m}^{\text{Order}}y_{m}}{C^{\text{Stock}}+(P+\sum_{m}C_{m}^{\text{Transport}}y_{m})\gamma }}$$ (7)

**Fig 1:**
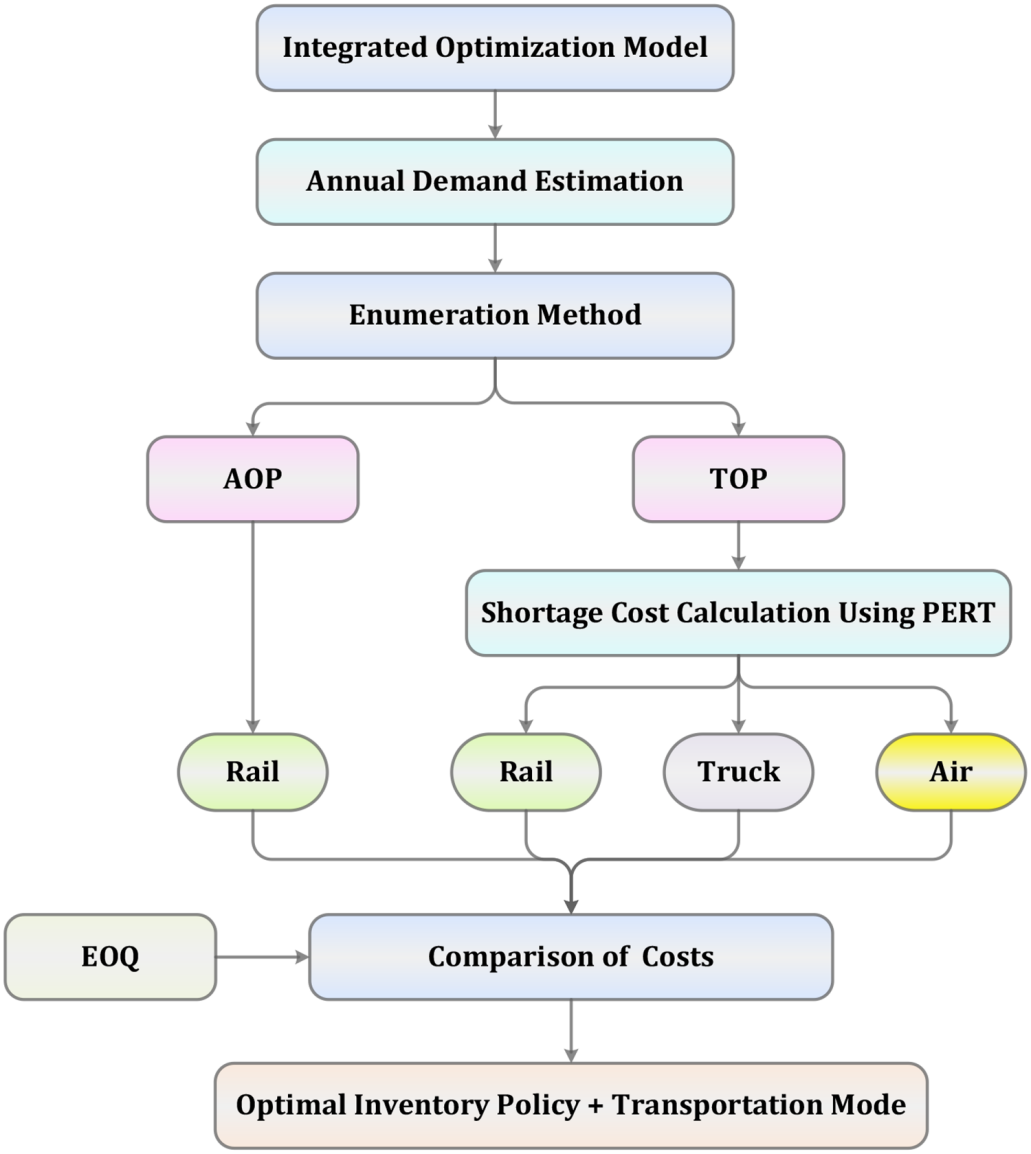
Solution framework.

By comparing the four corresponding objective values, the best inventory policy as well as the transportation mode can be obtained. The solution framework has also been depicted in Fig 1.

## Demand estimation method considering train maintenance strategy

In this section, we focus on the taxonomy of EMU maintenance spare parts and propose a demand estimation method for occasionally-replaced spare parts.

In production systems, the kinds of spare parts are wide-ranging and their number are always vast. As a result, developing inventory policies for each kind of spare parts is neither manageable nor labor-saving. It is both necessary and efficient to classify the spare parts into several categories so that a corresponding inventory policy can be developed for each category. In previous studies, researchers have addressed the maintenance parts classification problem from all perspectives. Marcello et al. [29] took inventory constraints, costs of lost production, safety and environmental objectives, strategies of maintenance adopted, logistics aspects of spare parts into account and defined spare parts classification with respect to multiple attributes. Molenaers et al. [30] proposed a spare parts classification method based on item criticality. In addition, ABC inventory analysis is also applied into classification of spare parts [31–33]. In this study, we present a novel classification method of EMU spare parts based on the maintenance strategy of China’s high-speed railway system as well as the parts’ replacement frequencies.

### EMU maintenance strategy in China’s high-speed railway system

In China’s high-speed railway system, the maintenance strategy is different from that of the conventional train service system. There are five maintenance grades for EMU trains according to the maintenance strategy. Among these, the first-grade and the second-grade maintenance are routine maintenance, and the remainder are called periodical maintenance or major maintenance (overhaul). The routine maintenance is performed at EMU inspection depots while the major maintenance is completed at EMU overhaul depots. From the first-grade to the fifth-grade, the maintenance content is increasingly complex. For example, in the first-grade maintenance, the depot staff only needs to do some routine inspection and cleaning work for EMU. However, in the second-grade maintenance, the staff not only have to complete the entire content of the first-grade maintenance but also must inspect some other assembly parts, such as bogies and pantographs. Furthermore, in the fifth-grade maintenance, the whole train body will be dismantled. Meanwhile, a wide range of spare parts will be replaced and the train surface will be repainted. According to the maintenance regulations, there is a mileage cycle and a time cycle correspond to each grade of maintenance. For example, the mileage cycle of the first-grade maintenance for CRH2 series EMU is 4,000 kilometers, whereas the time cycle is two days; the mileage cycle and the time cycle of the second-grade maintenance for CRH2 series EMU is 30,000 kilometers and 30 days, respectively. In other words, the CRH2 train needs to complete the first-grade maintenance at an inspection depot when it runs up to 4,000 kilometers or two days (depending on which cycle is met first). Similarly, when the train’s accumulative mileage reaches 30,000 kilometers or its accumulative time reaches 30 days, it must go to a depot to finish the second-grade maintenance. In addition, the maintenance service time is determined by the maintenance regulations. For example, the maintenance service time for the first-grade maintenance of CRH2 series EMU trains is one hour and fifty minutes, whereas the second-grade maintenance is eight hours.

### Taxonomy of EMU spare parts

The structure of an EMU is complex and its spare parts are of various kinds. The spare parts can be divided into three types according to the maintenance grades, i.e., inspection-depot-level parts, overhaul-depot-level parts and plant-level parts. Among them, the overhaul-depot-level parts and plant-level parts are both classified as overhaul parts.

1. The inspection-depot-level parts
  The inspection-depot-level parts are referred to those replaced or repaired during the first -grade, second-grade maintenance in inspection depots.
2. The overhaul-depot-level parts
  The overhaul level parts are those replaced or repaired during the third, fourth and fifth grade maintenance completed in overhaul depots.
3. The plant-level parts
  The plant-level parts are referred to those replaced and repaired when EMUs are overhauled in EMU plants or spare parts plants.

It is important to note that most EMU components are repaired based on their working conditions; that is, the parts of the EMU are not necessarily repaired or replaced every time the EMU arrives at the maintenance cycle. Thus, we can divide EMU spare parts into another two types: the definitely-replaced parts and the occasionally-replaced parts.

1. The definitely-replaced parts
  The definitely-replaced parts refer to the parts that must be repaired or replaced once the EMU meets the maintenance cycle, such as the strainer for air conditioning condensers, the gauze of the blower in the main transformer, the seal ring and the gaskets.
2. The occasionally-replaced parts
  The occasionally-replaced parts refer to the parts that need to be repaired or replaced with a certain probability once the EMU meets the maintenance cycle, such as the wheel set, absorber, the gear case and the brake disc.

### Average demand estimation for EMU occasionally-replaced parts

The demand for definitely-replaced parts can be accurately forecasted according to certain influential factors. However, forecasting the demand for OSP is difficult, because the demand is highly random and lacks of historical data. Even so, we can estimate the demand for OSP through certain factors, such as the number of EMUs, average daily travel distance and EMU’s maintenance cycle.

Let *N* denote the number of EMUs; *L* denotes the EMU’s average daily travel distance; *T*^Cycle^ denotes the EMU’s mileage cycle; *M* denotes the number of OSP equipped in one EMU train; *p* denotes the repair or replacement probability of OSP during overhaul. Recall that we have defined *T*^Interval^ as the average reorder interval in the TOP. In fact, this interval also refers to the average time interval of overhaul between two trains as the EMU depot orders parts only when trains are maintained. Therefore, the time interval can be expressed as follows:

$$T^{\text{Interval}}=\frac{T^{\text{Cycle}}}{L\cdot N}$$ (8)

Thus, the demand of OSP within one year can be expressed as follows:

$$D=\frac{365}{T^{\text{Interval}}}\cdot M\cdot p=\frac{365\cdot L\cdot M\cdot N\cdot p}{T^{\text{Cycle}}}$$ (9)

Without loss of generality, we only consider the maintenance mileage cycle of EMU trains in the mathematical formulation.

## Shortage cost accounting using PERT

The shortage of spare parts could have an adverse influence on the EMU maintenance process, such as prolonging maintenance time and causing shortage cost. The shortage cost is determined by two factors: shortage time and unit time shortage cost. These two factors will be analyzed in this section.

### Analysis of shortage time based on PERT

**Fig 2:**
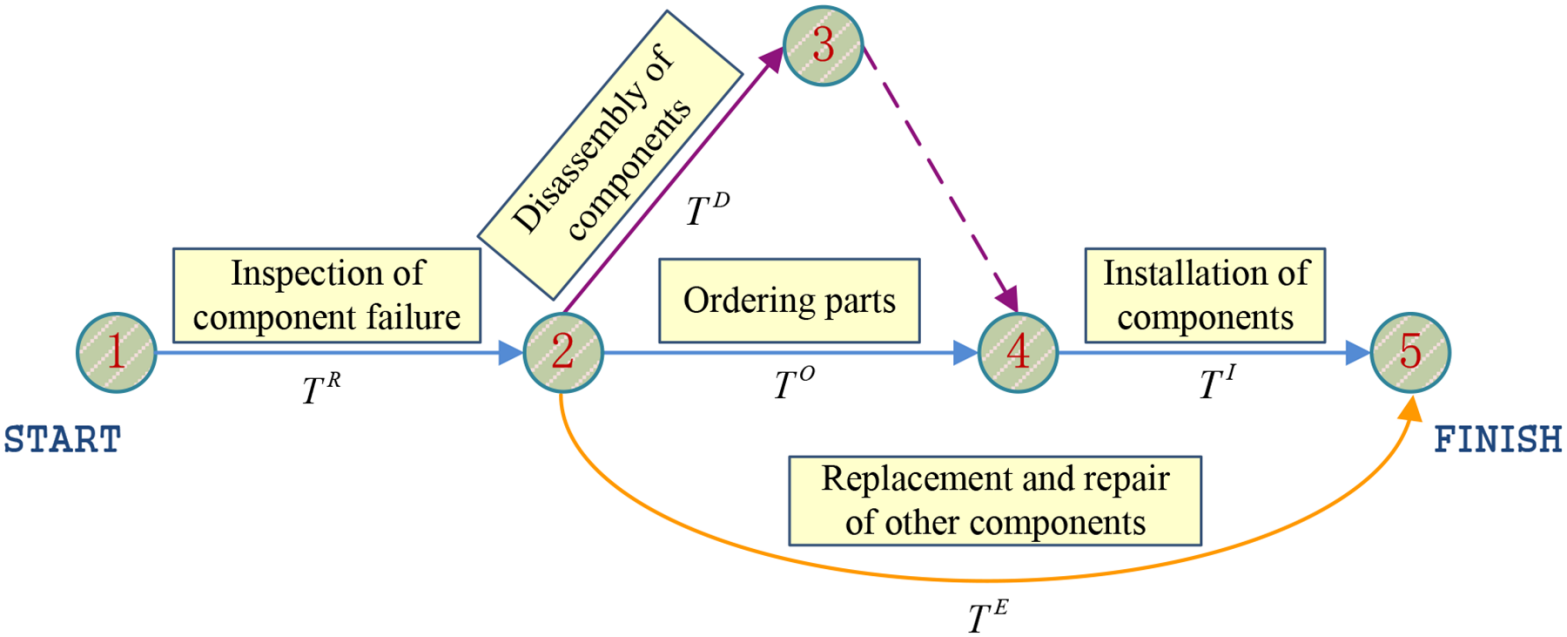
The PERT chart of EMU overhaul.

The shortage time of spare parts means the delay time of regular maintenance process, and it is not the equivalent of the order time (which contains the order processing time and transportation time). Since an EMU overhaul is a complicated project, the maintenance process and maintenance time of each part are not identical. Because of parallel operations, the shortage of a certain part is not necessarily going to influence the overall overhaul process. From the perspective of PERT, whether the shortage of spare parts will affect the overall overhaul process depends on whether the related maintenance operations are included in the critical path. To understand how the operation times affect the overhaul process, a PERT chart is constructed (see Fig 2).

As shown in Fig 2, each circle with a number in it represents a connecting point between the prior procedure (operation) and the next procedure. The names of the procedures are shown above arrow lines, and the times for the procedures are indicated below the arrow lines. When component failures are detected out, the EMU overhaul depot has to immediately order new parts to replace the faulted ones. During the contract signing and parts shipping period, parallel procedures such as component disassembly can be carried out. Meanwhile, disassembly, repair and replacement for other parts can also be implemented. Upon the arrival of new parts, installation procedure can be performed. As mentioned above, the total duration time of maintenance is determined by the length of the critical path *T*^Critical^. It is shown in the figure that there are three paths connecting the origin point ① and the destination point ⑤, i.e., ① → ② → ③ → ④ → ⑤, ① → ② → ④ → ⑤ and ① → ② → ⑤. The corresponding lengths of these paths are *T*^*R*^ + *T*^*D*^ +*T*^*I*^, *T*^*R*^ + *T*^*O*^ +*T*^*I*^ and *T*^*R*^ + *T*^*E*^, respectively. The length of critical path is equal to the maximum length of all paths in the PERT chart, which can be expressed as follows:

$$T^{\text{Critical}}=\text{max}(T^{R}+T^{D}+T^{I},T^{R}+T^{O}+T^{I},T^{R}+T^{E})$$ (10)

So, whether the parts procurement procedure belongs to the critical path depends not only on the duration itself but also on the duration of other procedures. This can be analyzed in three different situations.

1. *T*^*O*^ ≤ *T*^*D*^
  In this condition, the parts procurement procedure can never be included in the critical path, i.e., the shortage time *T*^Shortage^ equals zero.
2. *T*^*D*^ < *T*^*O*^ ≤ *T*^*E*^ −*T*^*I*^
  In this case, the critical path will be ① → ② →⑤. Although the ordering of parts might delay the component installation procedure, it has no influence on the total overhaul time. Consequently, the shortage time *T*^Shortage^ is also equal to zero.
3. *T*^*O*^ > *T*^*E*^ −*T*^*I*^
  On this occasion, the critical path is ① → ② → ④ → ⑤. The parts ordering procedure becomes a part of the critical path and thus partly determines the total overhaul time. Assuming that the regulated overhaul time is *T*^*R*^ + *T*^*E*^, then the shortage time will be *T*^Shortage^ = *T*^*O*^ + *T*^*I*^–*T*^*E*^.

Taking the above three cases into account, it can be concluded that there will be no stockout if *T*^*O*^ ≤ *T*^*E*^ −*T*^*I*^. On the contrary, shortage will appear if *T*^*O*^ > *T*^*E*^ −*T*^*I*^ and the shortage time is *T*^Shortage^ = *T*^*O*^ + *T*^*I*^–*T*^*E*^. In fact, parts ordering time *T*^*O*^ consists of order processing time *T*^Process^ and transportation time *T*^Transport^. However, online ordering technology has cut down the order processing time to a negligible level. Thus, spare parts ordering time could simply be equal to the transportation time; that is, *T*^*O*^ = *T*^Transport^. In this way, the shortage time can be re-expressed as follows:

$$\begin{array}{l} \begin{array}{l} T^{\text{Shortage}} \end{array} \end{array}=\{\begin{array}{ll} 0\; & T^{\text{Transport}}\leq T^{E}-T^{I}\\ T^{\text{Transport}}+T^{I}-T^{E} & T^{\text{Transport}}>T^{E}-T^{I} \end{array}$$ (11)

In our study, the unit of shortage time is day.

### Calculation of unit shortage cost

Because the transportation process for spare parts always consumes plenty of time, this could delay the EMU from returning into scheduled service. Though redundancy trains can serve as alternatives to recover the delay, there is still an opportunity cost for the delayed train caused by shortage of parts. When a train is absent from service, the ticket revenue cannot be collected. From this point of view, the unit day opportunity cost or the shortage cost is also stated as the shadow price of train ticket revenue for a single day. On the basis of these considerations, we can calculate the unit time shortage cost of spare parts as follows.

Let *ρ* denotes the average seat occupancy rate of one EMU train; *ω* denotes the fare rate (unit: CNY/passenger-kilometer, where CNY is short for China yuan); *B* denotes the train seating capacity; *α* denotes the net income ratio (the ratio of net income and ticket revenue). In the net income, operating expenses such as staff wages and abrasion of train have been deducted. Thus, the unit time shortage cost *C*^Shortage^ can be calculated by the following formula:

$$C^{\text{Shortage}}=\rho \cdot \omega \cdot \alpha \cdot B\cdot L$$ (12)

For example, if an EMU train can run 1,500 km one day, and the train has 800 seats with an average seat occupancy rate of 80% and a fare rate of 0.4 CNY/passenger-kilometer. We assume the net income ratio is 5%, the shortage cost equals 19,200 CNY/day. This is a considerable economical loss for railway companies.

## Case study

In this section, we focus on a real-world instance to illustrate the proposed methods. We first introduce the spare parts vendor and demander, and then calculate the total costs of inventory under the AOP and TOP under different transportation modes. Finally, comparisons are conducted with respect to the computational results.

### Case background

**Fig 3:**
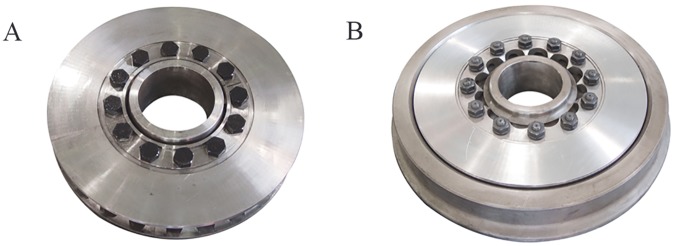
Brake discs of CRH2 series EMU. (A) The axle disc. (B) The wheel disc.

Without loss of generality, we select brake disc (including axle disc and wheel disc, see Fig 3) of the CRH2 series EMU (including six subseries: CRH2A, CRH2B, CRH2C, CRH2E, CRH380A, and CRH380AL) as overhaul OSP. As the OSP vendor, we choose China Qingdao Sifang Co., Ltd. (Sifang plant for short, formerly named SiFang Car & Locomotive Plant), and Shanghai EMU Depot as the demander.

Sifang plant is dedicated to the manufacture of high-speed trains, especially for the CRH2 series EMU, subway trains, and of course train spare parts. At present, Sifang plant has been one of the four largest EMU manufacturers of China, providing state-of-the-art technologies to support the rapid development of China high-speed railway. In contrast, as one of the largest EMU overhaul depots, Shanghai EMU Depot owned more than 300 EMU trains of all series at the beginning of 2015, and it consumes a large number of spare parts every year. The EMU Depot administrates several inspection depots (e.g., Shanghai Hongqiao Depot, Hangzhou Depot, and Nanjing Depot) that are located in the Yangtze River delta area and other areas in eastern China. These depots also have great demand for inspection- depot-level parts that are distributed by Shanghai Depot. However, the main focus of this paper is overhaul parts and thus the demands of the inspection depots are not considered.

### Calculation of average annual demand of brake discs

One EMU train consists of several motor cars and trailer cars. For instance, a CRH2A subseries EMU train is made up of four motor cars and four trailer cars (4M4T). The detailed make-up of CRH2 series EMU trains is shown in Table 1. There are 8 pairs of brake discs (i.e., 16 pieces of motor car wheel discs) for each motor car and 16 pairs of brake discs (including 16 pieces of axle discs and 16 pieces of trailer car wheel discs) for each trailer car. Therefore, the total number of brake discs equipped in a CRH2A train is 4×8+4×16 = 96 pairs. The number of brake discs for other subseries trains is also shown in Table 1. According to the relevant maintenance regulations, the repair or replacement of brake discs falls into the scope of third-grade maintenance with a mileage cycle of 600,000 km. To the best of our knowledge, the average daily running distance of EMU trains is approximately 1,500 km considering running time and depot dwell time (e.g., maintenance time). Shanghai EMU Depot has a total number of 136 CRH2 EMU trains, which means that the average third-grade maintenance time interval between two CRH2 EMU trains in Shanghai Depot is 600,000/(1,500×136)≈3 days. In other words, there are on average 365/3 = 122 trains that need to carry out third-grade maintenance in a one-year period. Given the number (proportion) of trains of each subseries (see Table 1), the annual maintenance demand for the trains are as follows: 52 for CRH2A, 9 for CRH2B, 41 for CRH2C, 8 for CRH2E, 7 for CRH380A, and 5 for CRH380AL. Noticing the number of brake discs equipped in one train, we can further compute the total number of brake discs to be inspected (not necessarily repaired or replaced) in one year, which equals to 52×96 + 9×192 + 41×80 + 8×192 + 7×80 + 5×144 = 12,816 pairs. Due to the fact that the brake disc belongs to the OSP, the disc is repaired or replaced only at a certain probability. Estimated by our collaborator at China Academy of Railway Sciences, the repaired or replaced probability for brake discs is approximately 0.01. Therefore, the average annual demand of brake discs for CRH2 series trains of the depot is 128 pairs.

**Table 1:** Information on CRH2 series EMU trains in Shanghai Depot.

EMU subseries	CRH2A	CRH2B	CRH2C	CRH2E	CRH380A	CRH380AL
**Number of trains**	58	10	46	9	8	5
**Train make-up**	4M4T	8M8T	6M2T	8M8T	6M2T	14M2T
**Number of brake discs (pairs/ train)**	96	192	80	192	80	144

### Ordering cost and unit transportation cost of brake discs

We assume that the supplier (i.e., Sifang plant) will provide a continuous supply of brake discs, which means that the EMU depot can order its required brake discs at any time. The EMU depot can choose either the ordinary strategy or the emergency strategy to order discs. Under the ordinary strategy, the depot can adopt the transport modes of railway and highway, whereas under the emergency strategy it can only choose the air transport mode. The ordering cost under the ordinary order strategy is 200 CNY/batch, whereas it is 300 CNY/batch under the emergency order strategy (see Table 2). The transportation costs and transit periods for different modes under different ordering strategies are shown in Table 2.

**Table 2:** Ordering cost with different ordering strategies in Shanghai EMU depot.

Ordering strategy	Transportation mode	Ordering cost (CNY/batch)	Transportation cost (CNY/ton)	Transportation time (days)
**Ordinary**	Rail^a^	200	292	9
	Truck^b^	200	950	3
**Emergency**	Air^c^	300	2000	1

Data source:

^a^ China Railway Customer Service Center (http://www.12306.cn)

^b^ Deppon Logistics Co., Ltd. (http://www.deppon.com/en/)

^c^ Shanghai Hangquan Air Freight Agency Co., Ltd. (http://hangquan.en.china.cn/)

### Total cost under the AOP

Since the charged weight of a pair of brake discs is approximately 70 kg, the transportation cost of single pair of discs is $C_{1}^{\text{Transport}}=292\times 0.07=20.44$ CNY. Given that the average purchase price of a pair of brake discs is 30,000 CNY, the annual storage cost is 300 CNY, and the annual interest rate is 6.0%, then the optimum order quantity of brake discs can be calculated according to Eq (7) as follows.

$$Q^{*}=\sqrt{\frac{2\times 200\times 128}{300+(30,000+20.44)\times 6.0\%}}=5$$ (13)

Thus, the annual order batches are 128/5≈26 with the average ordering cycle of 365/26 = 14 days. As mentioned before, the demand for brake discs is stochastic. According previous studies (e.g., Syntetos et al. [14]), we might as well assume that the demand *x* in the order lead time (approximately equal to the transit period, 9 days) follows a negative binomial distribution (NBD) whose mean and variance is *μ* = 3 and σ^2^ = 6, respectively. Thus, we can obtain the parameter *r* and parameter *p* for the NBD: *r* = 3 and *p* = 0.5. So the probability distribution function can be written by $\text{NBD}\left(x\right)=\left(\begin{array}{c} x+2\\ x \end{array}\right)\cdot 0.5^{x+3}$, where $\left(\begin{array}{c} x+2\\ x \end{array}\right)=\frac{\left(x+2\right)!}{x!2!}$. To ensure the inventory service level is larger than 95%, i.e., $\sum_{x=0}^{S}\text{NBD}\left(x\right)\geq 0.95$, the maximal stock level *S* in the order lead time should be no less than 6 pairs. Therefore, the reorder point is 6 pairs, and the safety stock is 6−3 = 3 pairs. Then we can calculate the total cost under the AOP according to Eq (5) as follows:

$$f(y_{1})=\frac{200\times 128}{5}+30,000\times 128+\left(3+\frac{5}{2}\right)\times \left[300+(30,000+20.44)\times 0.06\right]+20.44\times 128+\frac{0.06\times 30,000\times 128\times 9}{365}=3,864,974.16\;(\text{CNY)}$$ (14)

### Total cost under the TOP

According to the maintenance regulations of China railway, the third-grade maintenance duration time of CRH2 series EMU is 15 days, which means that the length of critical path of the maintenance process is 15 days. We assume that the inspection time of EMU components is *T*^*R*^ = 5 days, and then the repair or replacement time for spare parts except brake discs is *T*^*E*^ = 15–5 = 10 days. Meanwhile, we assume that the installation and debugging time for discs is *T*^*I*^ = 8 days. When the depot chooses the rail transport mode, the transportation time will be $T_{1}^{\text{Transport}}=9\text{ days}>T^{E}-T^{I}=2\text{ days}$ and the shortage will occur with a shortage time of 9–2 = 7 days. In contrast, when the road transport mode is adopted, the transportation time is $T_{2}^{\text{Transport}}=3\text{ days}>T^{E}-T^{I}=2\text{ days}$. In this case, the shortage time is 3 −2 = 1 day. However, if the depot selects the air transport mode, the transportation time is $T_{3}^{\text{Transport}}=1<T^{E}-T^{I}=2$ days and there are no stockouts.

For the shortage cost of one day, we can calculate it as follows. The fare rate of a CRH train is approximately 0.4 CNY/passenger·km, whereas the EMU’s average daily running distance is 1,500 km. The seating capacity of an 8-car CRH train is 600 passengers on average, and a 16-car train is 1,200 passengers. We can infer from Table 1 that the ratio of 8-cars trains to 16-cars trains is approximately 4:1 in Shanghai Depot. Therefore, the average seating capacity of a CRH2 series EMU is 600 × 0.8 + 1,200 × 0.2 = 720 passengers. We assume that the average seat occupancy of one train is 80% and the net income ratio is 5%. According to Eq (12), the shortage cost of a CRH2 series train is *C*^Shortage^ = *ρ*·*ω*·*α*·*B*·*L* = 80% × 0.4 × 0.05 × 720 × 1,500 = 17,280 CNY/day.

According to Eq (6), costs with different transportation modes can be calculated and we summarize the computational results in Table 3.

**Table 3:** Costs of different items under the TOP (in CNY).

Transportation mode	Ordering cost	Purchase cost	Transportation cost	In-transit cost	Shortage cost	Total cost
**Rail**	24,333.33	3,840,000	2,616.32	5,681.10	14,716,800	18,589,430.75
**Truck**	24,333.33	3,840,000	8,512.00	1,893.70	4,204,800	8,079,539.03
**Air**	36,500.00	3,840,000	17,920.00	631.23	0	3,895,051.23

### Comparison analysis

**Fig 4:**
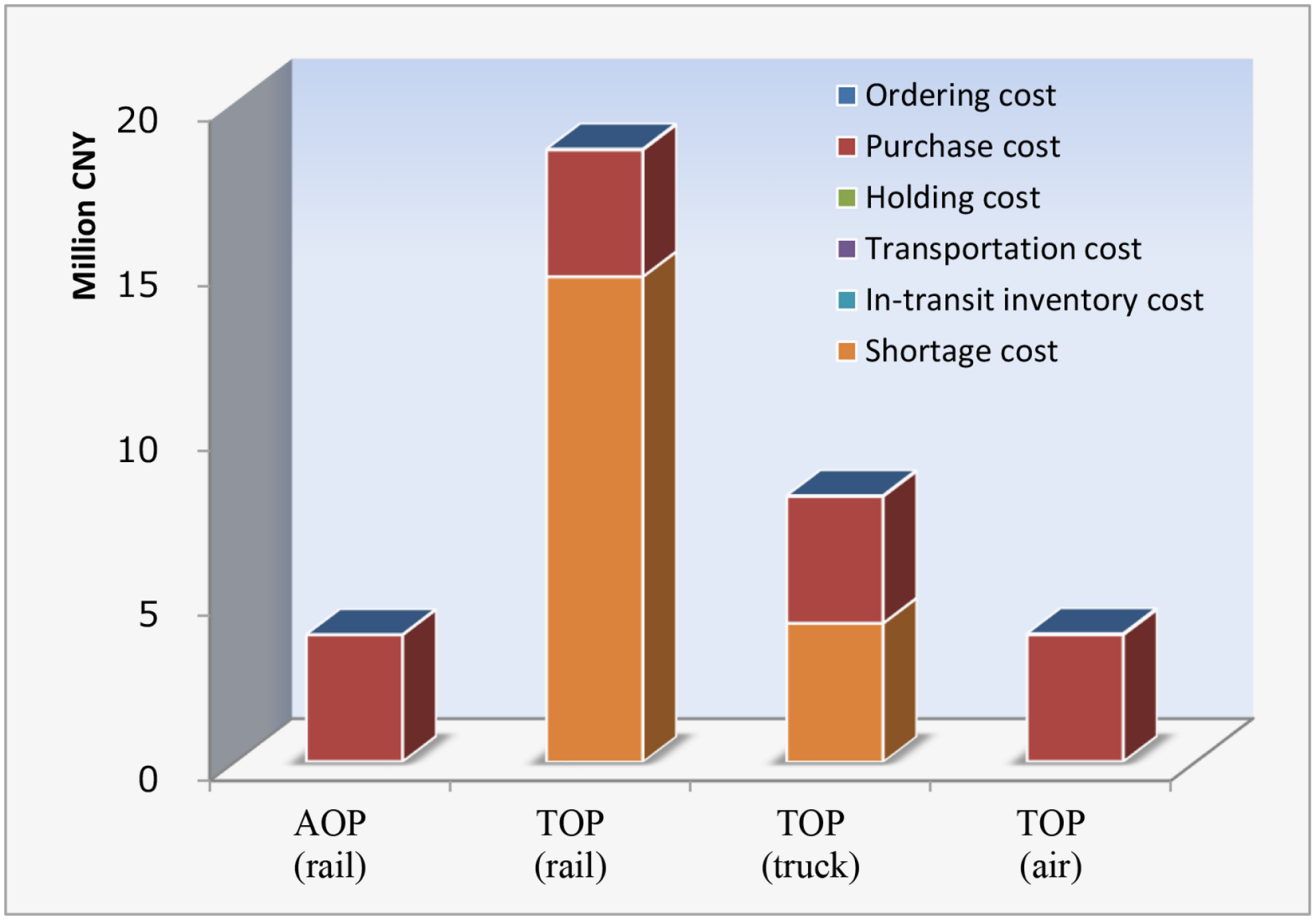
Composition of inventory costs under different inventory policies or transportation modes.

The composition of inventory costs under the AOP and the TOP (including three transport modes) is shown in Fig 4. We can conclude from Fig 4 that the total inventory costs depend mainly on the purchase cost as well as the shortage cost. In contrast, the share of the ordering cost, the inventory holding cost, the transportation cost and the in-transit inventory cost are so small that they are barely visible in Fig 4. Under the TOP, the most cost-saving solution is the emergency order strategy with the air transport mode (3,895,051.23 CNY), mainly because there is no stockout under this policy. However, the total costs under the AOP is 3,864,974.16 CNY, a decrease of 30,077.07 CNY compared with the TOP. If we do not consider the purchase cost (which is identical in both policies), then the total costs of inventory for the two strategies are 55,051.23 CNY and 24,974.16 CNY, respectively. Clearly, the latter has a 54.6% decrease compared with the former, which indicate that the AOP with the transport mode of railway is the best choice for Shanghai Depot. According to current practice in China railway system, when making a tradeoff between holding costs and the service level, the latter always has a priority. This is primarily because that the cancellation or delay of train services led by shortage of spare parts not only results in revenue loss, but also has negative impacts (e.g. reputation) on the railway operator. These negative impacts can be regarded as implicit costs and should be considered in future studies while quantification of implicit costs is often difficult.

Currently, the inventory policy for maintenance spare parts of high-speed trains is mainly manually developed by depot inventory managers in China high-speed railway system. The disadvantages of the manual method is quite distinct. Firstly, the manual method involves little optimization strategy. Instead, it is mainly based on the human experience. As a result, the computational results from the manual method are usually feasible solutions or so-called non-inferior solutions. However, by using the 0–1 programming based optimization method proposed in our paper, one can always obtain the optimal solution. From the viewpoint of optimality of final solutions (computational results), our approach is more effective than the manual method. Secondly, the manual method takes much longer time for inventory managers to develop inventory policies than that of our proposed approach. According to the filed investigation at Shanghai Depot, China, the inventory policy development process always requires couples of days of painstaking efforts by a team of highly experienced managers for thousands categories of spare parts. However, the proposed approach is easily implemented on computers given the input data, and the computational time is just a matter of seconds. Therefore, our solution approach is more efficient in terms of the computational speed. In all, the proposed approach is able to offer an effective and efficient decision support for depot inventory managers.

## Conclusions

With the rapid development of China high-speed railway, the maintenance demand for EMU trains has become increasingly strong. Spare parts are essential elements for carrying out maintenance activities, but due to their high-value characteristic and random demand, developing inventory policies for the spare parts is a challenging task. In this paper, we present a 0–1 programming model for the integrated inventory-transportation problem for EMU depots. To determine the annual demand, we propose a pragmatic demand estimation method for occasionally-replaced spare parts considering EMU maintenance strategies. Furthermore, we outline a calculation framework for shortage cost using the PERT. Finally, a real-world case study from Shanghai Depot is conducted to demonstrate the proposed method. The computational results indicate that the AOP with the rail transport mode is the best strategy for Shanghai Depot.

Our method offers an effective and efficient decision support for developing inventory policies for EMU depots. However, we roughly divide the inventory policies into the AOP and TOP. In practice, more sophisticated methods or policies could be applied. How to bridge this research gap is one of our future work. Furthermore, the following aspects can be also explored as interesting future research topics: (1) to extend our model to multi-vendor multi-type of spare parts scenarios; (2) to apply reasonable calculation schemes to each cost term with practical considerations; and (3) to extend our model to multi-echelon structure.

## References

[1] National Railway Administration of the People's Republic of China. 2015 Railway statistical bulletin (in Chinese) [2016-03-03]. http://www.nra.gov.cn/xwzx/zlzx/hytj/201603/t20160303_21466.shtml

[2] National Development and Reform Commission of the People's Republic of China. Mid- and long- term railway network planning (in Chinese) [2016-07-13]. http://www.sdpc.gov.cn/zcfb/zcfbtz/201607/t20160720_811696.html

[3] The Xinhua news agency. China's Standard high-speed EMU enters service (in Chinese) [2016-08-15]. http://news.xinhuanet.com/fortune/2016-08/15/c_1119392385.htm

[4] Sharif KI, Ibrahim JA, Udin ZM, Othman AA. Decision making of spare parts inventory based on risk quantification. International Journal of Supply Chain Management. 2016;5(3):96–9.

[5] Kutanoglu E, Lohiya D. Integrated inventory and transportation mode selection: A service parts logistics system. Transportation Research Part E: Logistics and Transportation Review. 2008;44(5):665–83. 10.1016/j.tre.2007.02.001

[6] Wu GD, Wang HS, Wen L, Li ZH. Research on spare parts management for EMU. Railway Locomotive & Car. 2011;31(2):38–41.

[7] Amini A, Ghodsi R. A linear mathematical model for a transportation-inventory problem in a two-stage supply chain with different types of fuels for vehicles. International Journal of Services and Operations Management. 2016;25(3):347–60.

[8] Wang WB, Syntetos AA. Spare parts demand: linking forecasting to equipment maintenance. Transportation Research Part E: Logistics and Transportation Review. 2011;47(6):1194–209. 10.1016/j.tre.2011.04.008

[9] Croston JD. Forecasting and stock control for intermittent demands. Operational Research Quarterly. 1972;23(3):289–303. 10.1057/jors.1972.50

[10] Kamath KR, Pakkala TPM. A Bayesian approach to a dynamic inventory model under an unknown demand distribution. Computers and Operations Research. 2002;29(4):403–22. 10.1016/S0305-0548(00)00075-7

[11] Wang M-C, Subba Rao S. Estimating reorder points and other management science applications by bootstrap procedure. European Journal of Operational Research. 1992;56(3):332–42. 10.1016/0377-2217(92)90316-2

[12] Watson BR. The effects of demand-forecast fluctuations on customer service and inventory cost when demand is lumpy. Journal of the Operational Research Society. 1987;38(1):75–82. 10.1057/jors.1987.9

[13] Burgin AT. The gamma distribution and inventory control. Journal of the Operational Research Society. 1975;26(3):507–25. 10.1057/jors.1975.110

[14] Syntetos AA, Babai MZ, Altay N. On the demand distributions of spare parts. International Journal of Production Research. 2012;50(8):2101–17. 10.1080/00207543.2011.562561

[15] Wang W. A stochastic model for joint spare parts inventory and planned maintenance optimisation. European Journal of Operational Research. 2012;216(1):127–39. 10.1016/j.ejor.2011.07.031

[16] Romeijnders W, Teunter R, van Jaarsveld W. A two-step method for forecasting spare parts demand using information on component repairs. European Journal of Operational Research. 2012;220(2):386–93. 10.1016/j.ejor.2012.01.019

[17] Kennedy WJ, Patterson JW, Fredendall LD. An overview of recent literature on spare parts inventories. International Journal of Production Economics. 2002;76(2):201–15. 10.1016/S0925-5273(01)00174-8

[18] Kocaga YL, Sen A. Spare parts inventory management with demand lead times and rationing. IIE Transactions. 2007;39(9):879–98. 10.1080/07408170601013646

[19] Selcuk B. An adaptive base stock policy for repairable item inventory control. International Journal of Production Economics. 2013;143(2):304–15. 10.1016/j.ijpe.2012.01.011.

[20] van Jaarsveld W, Dollevoet T, Dekker R. Improving spare parts inventory control at a repair shop. Omega. 2015;57:217–29. 10.1016/j.omega.2015.05.002

[21] Guajardo M, Rönnqvist M, Halvorsen AM, Kallevik SI. Inventory management of spare parts in an energy company. Journal of the Operational Research Society. 2015;66(2):331–41. 10.1057/jors.2014.8

[22] Topan E, Bayındır ZP, Tan T. Heuristics for multi-item two-echelon spare parts inventory control subject to aggregate and individual service measures. European Journal of Operational Research. 2017;256(1):126–38. 10.1016/j.ejor.2016.06.012

[23] Qu WW, Bookbinder JH, Iyogun P. An integrated inventory–transportation system with modified periodic policy for multiple products. European Journal of Operational Research. 1999;115(2):254–69. 10.1016/S0377-2217(98)00301-4

[24] Cardós M, García-Sabater JP. Designing a consumer products retail chain inventory replenishment policy with the consideration of transportation costs. International Journal of Production Economics. 2006;104(2):525–35. 10.1016/j.ijpe.2004.12.022

[25] Zhao Qh, Chen S, Leung SCH, Lai KK. Integration of inventory and transportation decisions in a logistics system. Transportation Research Part E: Logistics and Transportation Review. 2010;46(6):913–25. 10.1016/j.tre.2010.03.001

[26] Jha JK, Shanker K. An integrated inventory problem with transportation in a divergent supply chain under service level constraint. Journal of Manufacturing Systems. 2014;33(4):462–75. 10.1016/j.jmsy.2014.04.002

[27] Lee YCE, Chan CK, Langevin A, Lee HWJ. Integrated inventory-transportation model by synchronizing delivery and production cycles. Transportation Research Part E: Logistics and Transportation Review. 2016;91:68–89. 10.1016/j.tre.2016.03.017

[28] Kasilingam RG. Logistics and transportation: design and planning. London: Kluwer Academic Publishers; 1998.

[29] Marcello B, Andrea G, Roberto M. Multi-attribute classification method for spare parts inventory management. Journal of Quality in Maintenance Engineering. 2004;10(1):55–65. 10.1108/13552510410526875

[30] Molenaers A, Baets H, Pintelon L, Waeyenbergh G. Criticality classification of spare parts: a case study. International Journal of Production Economics. 2012;140(2):570–8. 10.1016/j.ijpe.2011.08.013

[31] Ramanathan R. ABC inventory classification with multiple-criteria using weighted linear optimization. Computers & Operations Research. 2006;33(3):695–700. 10.1016/j.cor.2004.07.014

[32] Hadi-Vencheh A. An improvement to multiple criteria ABC inventory classification. European Journal of Operational Research. 2010;201(3):962–5. 10.1016/j.ejor.2009.04.013

[33] Hatefi SM, Torabi SA, Bagheri P. Multi-criteria ABC inventory classification with mixed quantitative and qualitative criteria. International Journal of Production Research. 2013;52(3):776–86. 10.1080/00207543.2013.838328

